# How do we safely preserve ovaries in patients with cervical adenocarcinoma: risk factors and predictive models

**DOI:** 10.3389/fonc.2024.1464565

**Published:** 2024-10-30

**Authors:** Yunqiang Zhang, Yue Shi, Xuesong Xiang, Jingxin Ding, Keqin Hua

**Affiliations:** ^1^ Department of Gynecology Oncology, The Obstetrics and Gynecology Hospital of Fudan University, Shanghai, China; ^2^ Shanghai Key Laboratory of Female Reproductive Endocrine-Related Diseases, The Obstetrics and Gynecology Hospital, Fudan University, Shanghai, China

**Keywords:** cervical adenocarcinoma, ovarian metastasis, ovarian preservation, predictive model and machine learning, random forest

## Abstract

**Objective:**

To study and predict the risk of ovarian metastasis (OM) in patients with cervical adenocarcinoma (ADC).

**Methods:**

Patients with ADC who received surgical treatment from January 2015 to December 2022 in the Obstetrics and Gynecology Hospital of Fudan University were included in the study. Patients were further divided into OP (ovaries were preserved in surgery) and BSO (bilateral salpingo-oophorectomy) groups. For the patients who accepted BSO, 60% of the patients were randomly grouped into a training cohort, and predictive prognostic models were constructed with 10-fold cross-validation through random forest, LASSO, stepwise, and optimum subset analysis. The model with the highest area under receiver operator curve (AUC) was screened out in the testing cohort. The nomogram and its calibration curve presented the possibility of OM in future patients. The prognoses between the different populations were compared using Kaplan–Meier analysis. All data processing was carried out in R 4.2.0 software.

**Results:**

A total of 934 patients were enrolled in our cohort; 266 patients had their ovaries preserved and 668 patients had BSO according to the previous criteria reported The clinical safety with these criteria was secured, while the 5-year overall survival had no significant difference between the BSO and OP groups (p = 0.069), which suggested that the current criteria could be extended and are more precise. Four predictive models for ovarian metastasis by machine learning were constructed in our study, and the random forest model that obtained the highest AUC in both training and testing sets (0.971 for training and 0.962 for testing set) was taken as the best model. The optimal cut-off point of the ROC curve (specificity 99.5% and 90% sensitivity) was utilized to stratify the patients into high- and low-risk OM. Further comparing the survival curves of the high and low-OM risk groups, it was found that both DFS and OS were significantly prolonged in the low-risk group (p < 0.01). On the basis of this random forest model, a nomogram was used to calculate the OM risk, and the results were validated with calibration. The predictive model was further applied to the whole cohort (934 patients), and we identified the OM low-risk population (n = 822) and the patients with high risk who should be recommended for BSO (n = 112). No significant difference was found in the 5-year survival between the low-risk group with our model and the patients who already had ovaries preserved according to the previous criteria (n = 266).

**Conclusion:**

The predictive model constructed in our study could identify the low-risk population of OM in patients with ADC, which remarkably extended the number with the previous criteria, for whom we could potentially preserve ovaries to help improve their life quality.

## Introduction

Although screening with cytology and human papillomavirus (HPV) have undoubtedly led to a remarkable decline in cervical cancer burden, cervical cancer remains the fourth most common cancer among women worldwide. In young female of reproductive age, cervical cancer is consistently the second leading cause of cancer death in women aged 20–39 years according to Cancer Statistics, 2023 (1). The emergence of HPV vaccination could virtually eliminate HPV-related cervical cancer, most of which were squamous cervical cancer, and cervical adenocarcinoma (ADC) accounted for approximately 25% of all cervical cancer cases showing a significant increase, particularly among young women as to the rapidly growing use of HPV vaccine (2–4). ADC patients have considerable life expectancy as the 5-year overall survival (OS) of early-stage ADC is above 80% (5). Thus, ovarian preservation (OP) during surgery seems crucial for younger patients to conserve ovarian endocrine function and fertility. However, considering the medical safety, OP during surgery has been always controversial for ADC patients. Some previous studies recommended that ADC patients should be routinely ovariectomized as the incidence of OM in ADC was reported to be above 10% (6, 7) Yet, more evidence arose to support OP in selective ADC patients (8–11) and the selection criteria for OP reported in 2015 (**Table 1**) (12). OP surgery for ADC patients has been widely performed in accordance with the criteria in our center since 2015. Subsequent pathological analysis of the removed ovaries from patients who did not meet the criteria revealed that many of them have no OM, which might indicate that the criteria should be modified, and a predictive model is needed to extend the range involving more ADC patients to preserve ovaries.

**Table 1:** Detailed selection criteria for OP reported (13).

Preoperative patients’ characteristics
–Age ≤45 years
–Patients who desire to retain ovarian function
–No familial predisposition to ovarian cancer
Tumor characteristics based on clinical and imaging evidence
–FIGO stage ≤IB2
–Size of the tumor ≤4 cm
–No parametrial invasion
–No corpus invasion
–No deep stromal invasion
–No evidence of lymph node metastasis (CT scan or PET scan)
–No lymphatic vascular space invasion (LVSI)
Intraoperative findings
–No extra-uterine spread
–No lymph node metastasis
–Normal appearance of ovaries

However, studies on OP in cervical adenocarcinoma since 2015 have focused exclusively upon the clinical safety and potential high-risk factors causing ovarian metastasis, while no new criteria or predictive models were constructed to our knowledge (8, 13–16). Therefore, we collected and studied the characteristics of ADC patients treated in the past 7 years in our center to build and validate a reliable model for OM in cervical adenocarcinoma, subsequently to more accurately preserve ovaries in ADC patients and largely improve their life quality. This was the first time a model for predicting ovarian metastasis of cervical adenocarcinoma was constructed using the machine learning method, which can be practically utilized in real clinic scenario.

## Materials and methods

### Study setting and design

This was a retrospective study conducted from January 2015 to December 2022 in the Obstetrics and Gynecology Hospital of Fudan University in Shanghai, China, which is the largest medical center for cervical cancer diagnosis and treatment in China with our patients covering 34 states all over China.

### Study population

Cervical cancer patients at early stage [stages IA–IIA, restaged according to FIGO 2019 (17)], surgically treated between January 2015 and December 2022, were reviewed with informed consent. The study was approved by the medical ethics committee of the Obstetrics and Gynecology Hospital of Fudan University (Ob&Gyn Hospital) (No. 2022-123) on 31 August 2022 and conducted in accordance with the Declaration of Helsinki. Patients with stage IA1 disease were treated through hysterectomy. Modified radical hysterectomy (18) was performed on patients with FIGO stage IA1 coupled with positive margin or LVSI and FIGO stage IA2. Radical hysterectomy was performed on patients with FIGO stages IB to IIA2 cervical cancer. Pelvic lymphadenectomy ± para-aortic lymphadenectomy was performed on all except for those with FIGO stage IA1 disease. Fallopian tubes were routinely removed and whether or not to remove the ovaries was decided according to the criteria shown in **Table 1**.

### Explanatory and outcome variables

Both clinical and pathological data were obtained from medical records. The explanatory clinicopathological variables included patient age, pre- and post-surgery pathological diagnosis (WHO 2014), tumor size, lymph node metastasis (LNM), location of LNM, depth of stromal invasion, LVSI status, parametrial invasion, uterine corpus invasion (UCI), surgical margin, vaginal involvement, fallopian tube metastasis, OM, FIGO stage (2019), and treatment modalities. The outcome variables included OM according to post-surgery pathology, as well as overall survival (OS) and disease-free survival (DFS) measured from the date of surgery to recurrence/death or the last time patients were censored at follow-up. The end point of follow-up was December 2023, and the date of recurrence/death was obtained from our hospital’s follow-up records.

### Descriptive statistics and variable selection

For continuous covariates, we employed receiver operating characteristic (ROC) curves to determine cut-off points for classifying patients into high- and low-risk groups. This approach, facilitated by the “ROCR” R package, allowed us to identify thresholds that optimally discriminated between patients with and without ovarian metastasis. Categorical variables were compared using Chi-squared and Fisher’s exact tests, implemented through the “tableone” R package, to ensure that our baseline characteristics were balanced across risk groups.

### Model construction, selection, and calibration

The 668 patients who underwent bilateral salpingo-oophorectomy (BSO) were randomly divided into training and testing cohorts (including 401 and 267 patients, respectively) using “caret” R package. To identify potentially significant factors affecting ovarian metastasis risks, we performed univariate logistic regression analyses on the training cohort. Factors with p-values < 0.1 were considered candidates for inclusion in the multivariate models. Then, we employed multiple methods to construct risk scores in the training cohort, including Least Absolute Shrinkage and Selection Operator (LASSO) regression, stepwise multivariate logistic analysis, optimum subset logistic regression, and random survival forest analysis. Each method was chosen for its unique strengths in handling high-dimensional data, avoiding overfitting, and capturing complex interactions. The method that yielded the highest area under the curve (AUC) of the ROC in the testing cohort was selected as the final model for ovarian metastasis risk prediction. The cutoff point determined by the ROC was used to evaluate whether our patients could preserve ovaries or not in our predictive model. After that, the nomogram and related calibration curves were established based on a new model to present the probability of ovarian metastasis in future patients.

### Software and tools

All data processing and analyses were conducted using R 4.2.0 software leveraging a range of specialized packages including “glmnet,” “caret,” “randomForest,” “My.stepwise,” “forestploter,” “forestplot,” “bestglm,” “leaps,” “genefilter,” “Hmisc,” “ISLR,” “rms,” “regplot,” and “ROCR.” These packages offer powerful and flexible tools for statistical modeling, visualization, and interpretation enabling us to conduct our analyses with precision and efficiency.

## Results

### Patient characteristics

**Figure 1:**
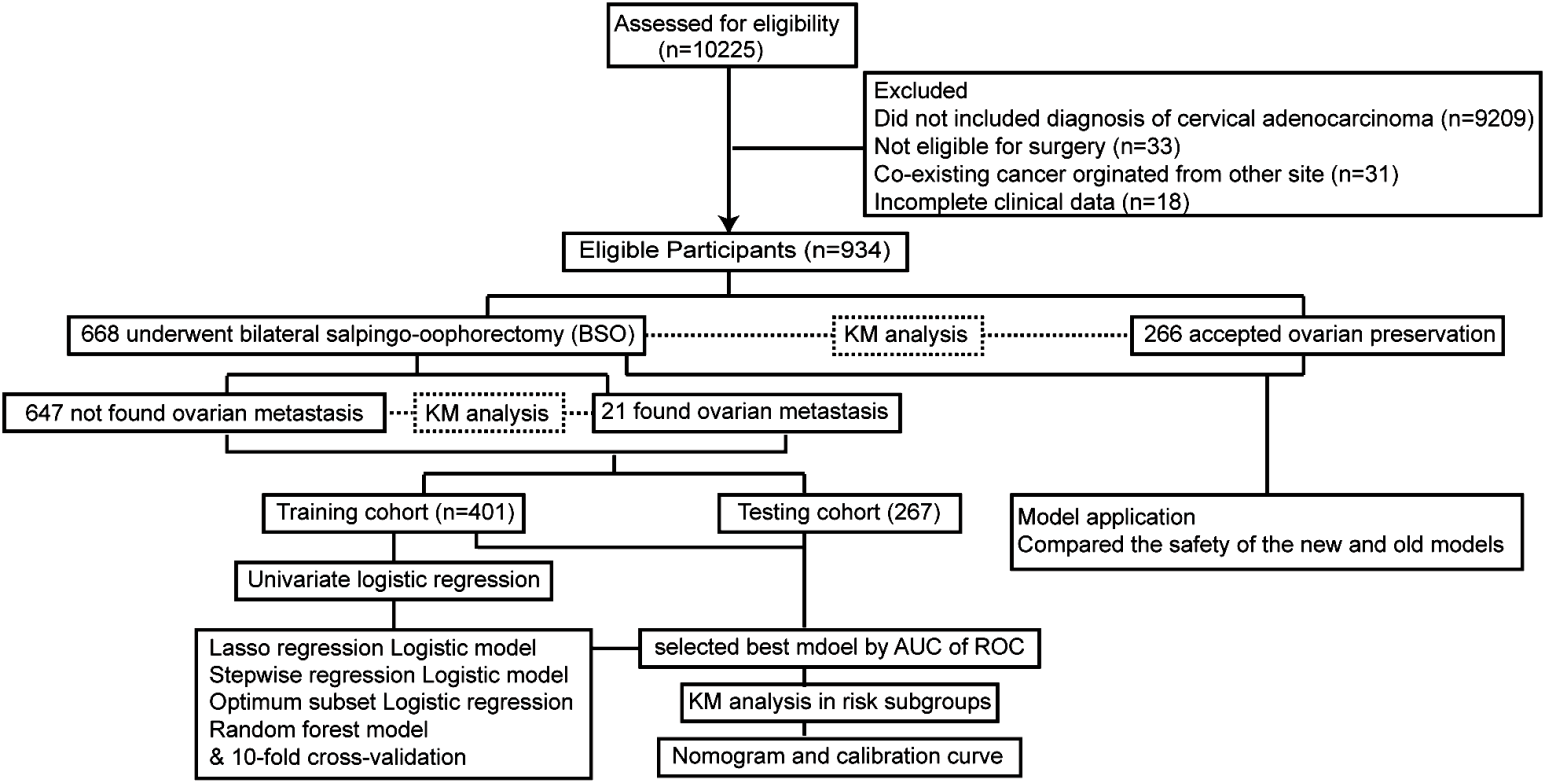
Flowchart of the study process.

A total of 934 patients who were screened with inclusion criteria were finally enrolled in our study (**Figure 1**), with an average age of 46.32 ± 9.53 years (median: 45.50 years; range: 22–74 years), among whom BSO was performed in 668 cases and OP in 266 patients based on the criteria (**Table 1**) (12). Patients who had OP were younger than those who had their ovaries removed (37.42 ± 5.79 years *vs.* 50.04 ± 8.23 years). Patients in the BSO group possessed more risk factors as expected, such as advanced stage, LNM, LVSI, DMI, parametric invasion, and UCI. The results of the univariate analysis in **Table 2** also confirmed that ovarian metastasis should be associated with these risk factors, further supporting the validity of the selection criteria. Besides, different pathology types may have different risks of ovarian metastasis, while histopathology type was not included in the previous criteria. The association of ovarian metastasis with different histopathology types was analyzed (**Table 3**) and further classified into high- and low-risk histopathology types with a cutoff risk of 10%. Among the high-risk pathologic type patients, only 4/74 had ovaries preserved (**Table 2**), and the rate of ovarian metastasis for those who had their ovaries removed was as high as 17.14% (**Table 4**).

**Table 2:** Clinicopathologic comparison in patients with ovarian preservation (OP) or bilateral salpingo-oophorectomy (BSO) during surgical treatment.

	Total	BSO	OP	p-Value
		668	266	
**Age (n, %)**				<0.001
≤50	640 (68.5)	376 (56.3)	264 (99.2)	
>50	294 (31.5)	292 (43.7)	2 (0.8)	
**Histological classification (n, %)**				<0.001
Low risk	858 (92.1)	598 (89.5)	260 (98.5)	
High risk	74 (7.9)	70 (10.5)	4 (1.5)	
**FIGO stage (n, %)**				0.002
I	679 (72.7)	462 (69.2)	217 (81.6)	
II	85 (9.1)	67 (10.0)	18 (6.8)	
III	163 (17.5)	133 (19.9)	30 (11.3)	
IV	7 (0.7)	6 (0.9)	1 (0.4)	
**Tumor size (n, %)**				0.075
≤4 cm	717 (83.1)	510 (81.6)	207 (87.0)	
>4 cm	146 (16.9)	115 (18.4)	31 (13.0)	
**LNM (n, %)**				0.006
Negative	726 (80.8)	508 (78.4)	218 (87.2)	
Pelvic LN	104 (11.6)	82 (12.7)	22 (8.8)	
Common iliac LN	50 (5.6)	40 (6.2)	10 (4.0)	
Paraaortic LN	18 (2.0)	18 (2.8)	0 (0.0)	
**DMI (n, %)**				<0.001
Superficial 1/3	482 (51.6)	304 (45.5)	178 (66.9)	
Middle 1/3	29 (3.1)	20 (3.0)	9 (3.4)	
Deep 1/3	389 (41.6)	315 (47.2)	74 (27.8)	
Whole cervical layer	34 (3.6)	29 (4.3)	5 (1.9)	
**LUSI (n, %)**				<0.001
Negative	727 (77.8)	497 (74.4)	230 (86.5)	
Positive	207 (22.2)	171 (25.6)	36 (13.5)	
**LVSI (n, %)**				0.001
Negative	583 (62.4)	392 (58.7)	191 (71.8)	
Sporadic	334 (35.8)	263 (39.4)	71 (26.7)	
Extensive	17 (1.8)	13 (1.9)	4 (1.5)	
**Parametrial involvement (n, %)**				0.002
Negative	818 (91.9)	579 (90.0)	239 (96.8)	
Positive	72 (8.1)	64 (10.0)	8 (3.2)	
**Incision margin (n, %)**				0.006
Negative	895 (95.8)	632 (94.6)	263 (98.9)	
Positive	39 (4.2)	36 (5.4)	3 (1.1)	
**Vaginal invasion (n, %)**				<0.001
Negative	832 (89.1)	577 (86.4)	255 (95.9)	
Positive	102 (10.9)	91 (13.6)	11 (4.1)	
**Fallopian metastasis (n, %)**				0.033
Negative	825 (96.7)	641 (96.0)	184 (99.5)	
Positive	28 (3.3)	27 (4.0)	1 (0.5)	
**Adjuvant chemotherapy (n, %)**				<0.001
No	413 (46.4)	264 (41.6)	149 (58.4)	
Yes	477 (53.6)	371 (58.4)	106 (41.6)	
**Adjuvant radiotherapy (n, %)**				<0.001
No	457 (51.5)	294 (46.3)	163 (64.7)	
Yes	430 (48.5)	341 (53.7)	89 (35.3)	

FIGO, International Federation of Gynecology and Obstetrics; LNM, lymph node metastasis; LUSI, lower uterine segment involvement; DMI, depth of myometrial invasion; LVSI, lymph vascular space invasion.

**Table 3:** The OM distribution in different pathological subtypes of ADC.

Pathological type (WHO 2014)	OM	
	Yes	No
Low risk		
Villoglandular	0 (0.0)	3 (100.0)
Endometrioid	0 (0.0)	5 (100.0)
Intestinal type	0 (0.0)	21 (100.0)
Clear cell type	0 (0.0)	8 (100.0)
Mixed with neuroendocrine	0 (0.0)	15 (100.0)
Usual adenocarcinoma type	3 (1.18)	252 (98.82)
Mucinous, NOS	1 (1.30)	76 (98.70)
Adenosquamous	4 (1.87)	210 (98.13)
High risk		
Gastric type	5 (11.90)	37 (88.10)
Serous	2 (15.38)	11 (84.62)
MDA	4 (28.57)	10 (71.43)
Signet ring cell type	1 (100.0)	0 (0.0)

**Table 4:** Clinicopathologic characteristics associated with OM in the BSO group.

	668	BSO group		
		No OM	OM	p-Value
		647	21	
**Age (n, %)**				0.138
≤50	376 (56.3)	368 (56.9)	8 (38.1)	
>50	292 (43.7)	279 (43.1)	13 (61.9)	
**Histological classification (n, %)**				<0.001
Low risk	598 (89.5)	589 (91.0)	9 (42.9)	
High risk	70 (10.5)	58 (9.0)	12 (57.1)	
**FIGO stage (n, %)**				<0.001
I	462 (69.2)	457 (70.6)	5 (23.8)	
II	67 (10.0)	65 (10.0)	2 (9.5)	
III	133 (19.9)	123 (19.0)	10 (47.6)	
IV	6 (0.9)	2 (0.3)	4 (19.0)	
**Tumor size (n, %)**				0.025
≤4 cm	510 (81.6)	498 (82.3)	12 (60.0)	
>4 cm	115 (18.4)	107 (17.7)	8 (40.0)	
**LNM (n, %)**				<0.001
Negative	508 (78.4)	502 (79.8)	6 (31.6)	
Pelvic LN	82 (12.7)	77 (12.2)	5 (26.3)	
Iliac LN	40 (6.2)	36 (5.7)	4 (21.1)	
Paraaortic LN	18 (2.8)	14 (2.2)	4 (21.1)	
**DMI (n, %)**				<0.001
Superficial 1/3	304 (45.5)	302 (46.7)	2 (9.5)	
Middle 1/3	20 (3.0)	20 (3.1)	0 (0.0)	
Deep 1/3	315 (47.2)	301 (46.5)	14 (66.7)	
Whole cervical layer	29 (4.3)	24 (3.7)	5 (23.8)	
**LUSI (n, %)**				<0.001
Negative	497 (74.4)	493 (76.2)	4 (19.0)	
Positive	171 (25.6)	154 (23.8)	17 (81.0)	
**LVSI (n, %)**				<0.001
Negative	392 (58.7)	385 (59.5)	7 (33.3)	
Sporadic	263 (39.4)	255 (39.4)	8 (38.1)	
Extensive	13 (1.9)	7 (1.1)	6 (28.6)	
**Parametrial involvement (n, %)**				<0.001
Negative	579 (90.0)	569 (91.0)	10 (55.6)	
Positive	64 (10.0)	56 (9.0)	8 (44.4)	
**Incision margin (n, %)**				<0.001
Negative	632 (94.6)	621 (96.0)	11 (52.4)	
Positive	36 (5.4)	26 (4.0)	10 (47.6)	
**Vaginal invasion (n, %)**				<0.001
Negative	577 (86.4)	569 (87.9)	8 (38.1)	
Positive	91 (13.6)	78 (12.1)	13 (61.9)	
**Fallopian metastasis (n, %)**				<0.001
Negative	641 (96.0)	631 (97.5)	10 (47.6)	
Positive	27 (4.0)	16 (2.5)	11 (52.4)	
**Adjuvant chemotherapy (n, %)**				0.001
No	264 (41.6)	264 (42.7)	0 (0.0)	
Yes	371 (58.4)	354 (57.3)	17 (100.0)	
**Adjuvant radiotherapy (n, %)**				0.031
No	294 (46.3)	291 (47.1)	3 (17.6)	
Yes	341 (53.7)	327 (52.9)	14 (82.4)	

### OM and survival analysis in patients with ADC

**Figure 2:**
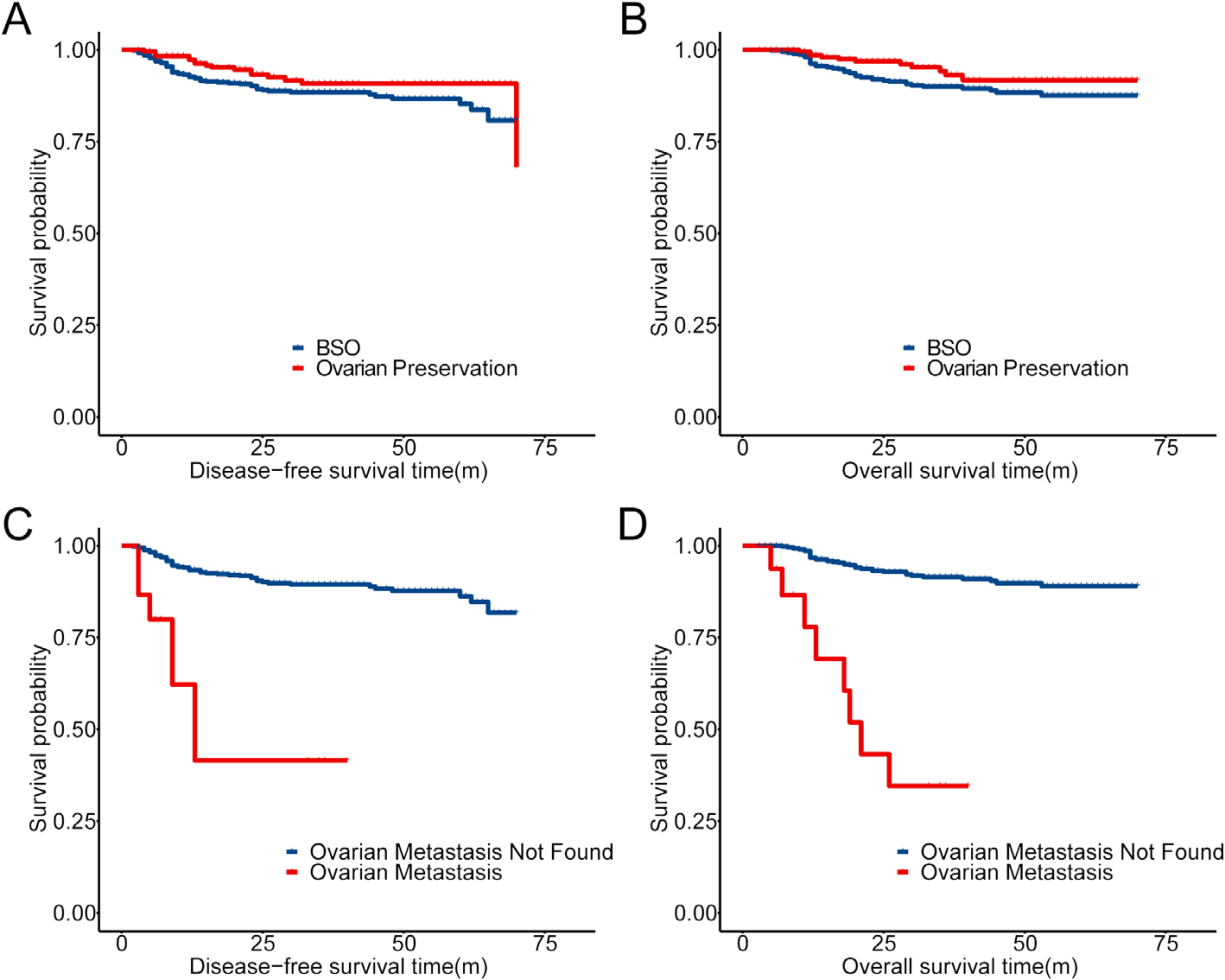
Prognostic comparison in our cohort with Kaplan–Meier (KM) analysis. **(A, B)** Prognostic comparison of patients with OP and BSO in the cohort (934 patients). **(C, D)** Prognostic comparison of patients with ovarian metastasis or not in 668 patients who accepted BSO.

The overall 5-year OS among our cohort with 934 patients was 88.8%. In patients with adnexectomy, the total rate of OM was 3.14%. For patients with OM, all of them received adjuvant chemotherapy, and 82.4% had radiation therapy (RT) (**Table 4**). Compared to patients without OM, the 5-year DFS (**Figure 2C**) and OS (**Figure 2D**) of patients with OM decrease significantly, which indicated that surgery plus adjuvant chemoradiotherapy might not improve the outcome of patients with OM. On the other hand, there was no significant difference in 5-year DFS and OS between the BSO and OP groups (p = 0.09 and 0.069, **Figures 2A, B**).

### Construction of the predictive model for OM in patients with ADC

**Figure 3:**
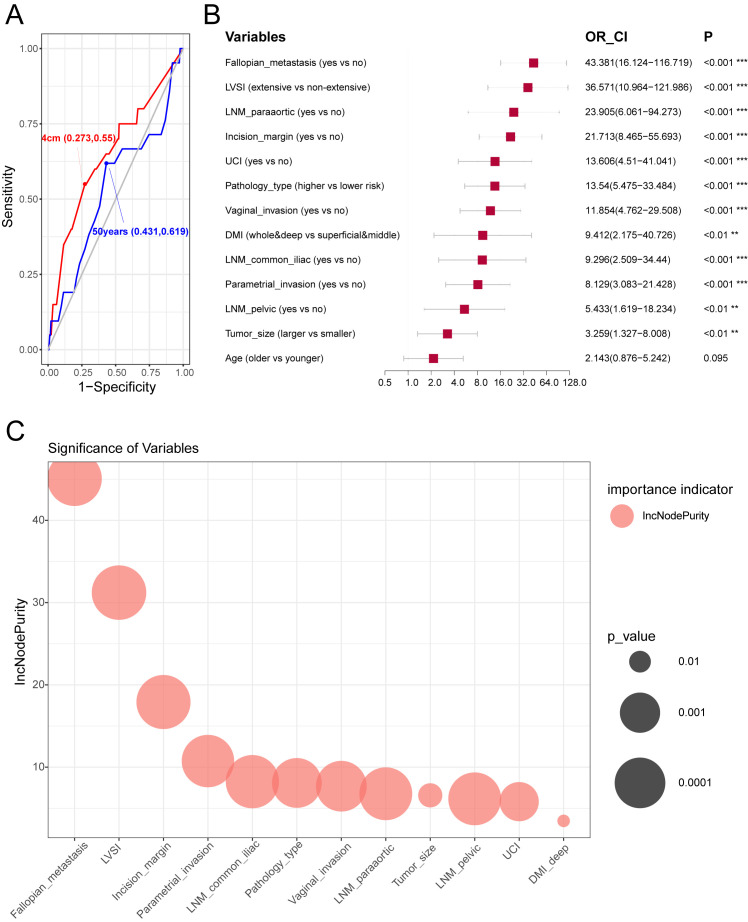
The optimal cut-off points of clinicopathological variables on prognosis and construction of models to predict ovarian metastasis. **(A)** Receiver operator curve (ROC) of age and tumor maximal diameter in predicting ovarian metastasis. The optimal cut-off points were marked on the plot. **(B)** Univariate logistic regression of clinicopathological variables related to ovarian metastasis in the training cohort. Hazard ratio, and 95% confidence interval are shown in forest plot using the “forestplot” R package. **(C)** The significant order of variables related to ovarian metastasis using the random forest model in the training cohort. “%IncMSE” means “increase in mean squared error (%),” and the randomForest R package was used.

Patients who underwent BSO were divided it into training and test sets for machine learning of ovarian metastasis at a ratio of 6:4. No difference was found between these two sets regarding the baseline clinicopathological characteristics (**Table 5**). Clinicopathological variables were analyzed for association with OM (**Figure 3**), and cutoff value for age and tumor size as continuous variables were defined by ROC curves at 50-year old and 4 cm, respectively (**Figure 3A**). Variables significantly associated with OM screened by univariate regression analysis in the training set included fallopian tube metastasis, LVSI (categorized as extensive and non-extensive as no significant difference was found between no LVSI and sporadic LVSI), DMI, LNM metastasis, incision margin status, UCI, pathological type, and tumor size. The two critical variables associated with OM fallopian tube metastasis and extensive LVSI status were proven by the OR value generated by univariate analysis (**Figure 3B**) and random forest model analysis.

**Table 5:** Clinicopathologic characteristics of the training and testing population in 668 patients who accepted BSO.

	n = 668	Cohort		
		Training	Testing	p-Value
		401	267	
**Age (n, %)**				0.847
≤50	376 (56.3)	224 (55.9)	152 (56.9)	
>50	292 (43.7)	177 (44.1)	115 (43.1)	
**Histological classification (n, %)**				0.902
Low risk	598 (89.5)	358 (89.3)	240 (89.9)	
High risk	70 (10.5)	43 (10.7)	27 (10.1)	
**Tumor size (n, %)**				0.646
≤4 cm	449 (71.8)	271 (72.7)	178 (70.6)	
>4 cm	176 (28.2)	102 (27.3)	74 (29.4)	
**LNM (n, %)**				0.574
No	508 (78.4)	309 (79.2)	199 (77.1)	
Pelvic	82 (12.7)	51 (13.1)	31 (12.0)	
Common iliac	40 (6.2)	21 (5.4)	19 (7.4)	
Paraaortic	18 (2.8)	9 (2.3)	9 (3.5)	
**DMI (n, %)**				0.604
Superficial and middle	324 (48.5)	190 (47.4)	134 (50.2)	
Deep	315 (47.2)	195 (48.6)	120 (44.9)	
Whole	29 (4.3)	16 (4.0)	13 (4.9)	
**LUSI (n, %)**				0.835
Negative	497 (74.4)	300 (74.8)	197 (73.8)	
Positive	171 (25.6)	101 (25.2)	70 (26.2)	
**LVSI (n, %)**				0.456
Non-extensive	655 (98.1)	395 (98.5)	260 (97.4)	
Extensive	13 (1.9)	6 (1.5)	7 (2.6)	
**Parametrial involvement (n, %)**				0.558
Negative	579 (90.0)	344 (89.4)	235 (91.1)	
Positive	64 (10.0)	41 (10.6)	23 (8.9)	
**Incision margin (n, %)**				0.698
Negative	632 (94.6)	381 (95.0)	251 (94.0)	
Positive	36 (5.4)	20 (5.0)	16 (6.0)	
**Vaginal invasion (n, %)**				0.508
Negative	577 (86.4)	343 (85.5)	234 (87.6)	
Positive	91 (13.6)	58 (14.5)	33 (12.4)	
**Fallopian metastasis (%)**				0.085
Negative	641 (96.0)	380 (94.8)	261 (97.8)	
Positive	27 (4.0)	21 (5.2)	6 (2.2)	
**Ovarian metastasis (%)**				0.34
Negative	647 (96.9)	391 (97.5)	256 (95.9)	
Positive	21 (3.1)	10 (2.5)	11 (4.1)	

Our data showed that no significant difference was found between no LVSI and sporadic LVSI affecting OM; also, no difference was found between the superficial infiltration and middle one-third invasion of the cervix in affecting OM, so we categorized these two parameters into extensive LVSI vs. non-extensive LVSI, and deep + whole muscular invasion vs. superficial + middle one-third muscular infiltration of the cervix for further analysis.

**Figure 4:**
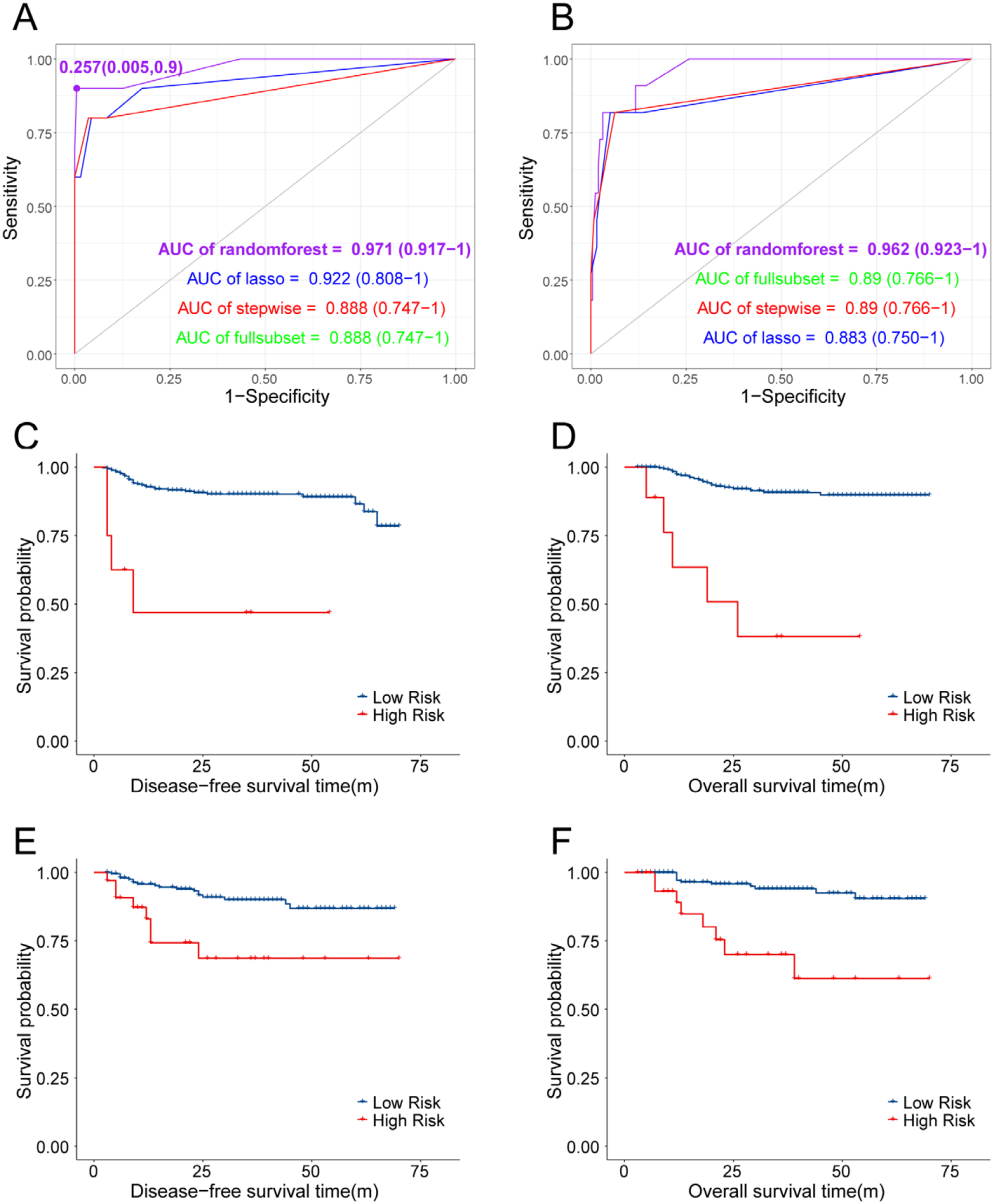
**(A, B)** ROC of different predictive models established in predicting OM and AUC were compared. **(C, D)** KM curves of DFS and OS between patients predicted with high-risk and low-risk of OM in training cohort. **(E, F)** KM curves of DFS and OS between patients predicted with high-risk and low-risk of OM in testing cohort.

**Figure 5:**
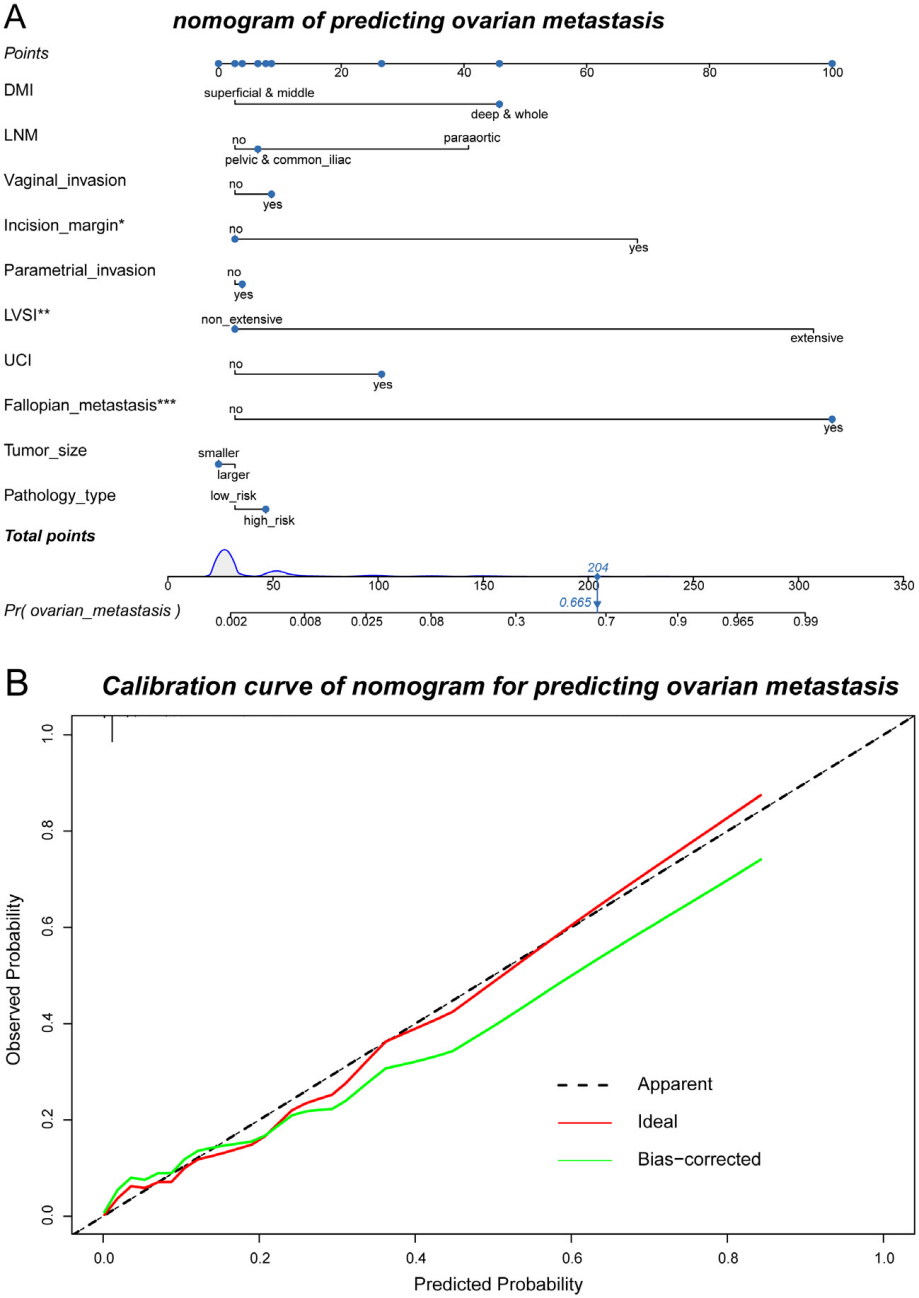
Nomogram of predicting ovarian metastasis and its calibration curves. **(A)** Nomogram of the randomForest model to predict the possibility of ovarian metastasis. **(B)** Calibration curve of the nomogram used for predicting ovarian metastasis.

Machine learning was performed with the following four methods: random forest, stepwise regression, LASSO, and optimal subset. The random forest model obtained the highest AUC in both the training and testing sets (0.971 for training set and 0.962 for testing set), considered to be the optimal predictive model. Also, the OM high- and low-risk subgroups were divided according to the optimal cutoff point of the ROC curve with its specificity at 99.5% and sensitivity at 90% (**Figures 4A, B**). Both DFS and OS differed significantly with this categorization (**Figures 4C, D**), which was further validated in the testing set (**Figures 4E, F**) with ovarian metastasis rate as high as 25.00% in the high-risk group and 0.44% in the low-risk group. On the basis of this random forest model, we further utilized nomogram to predict OM risk and validated the model with calibration curves (**Figure 5**). Details of the other three methods (stepwise regression, LASSO, and optimal subset) are displayed in **Supplementary Figure S1**.

### Security verification of the predictive model

**Figure 6:**
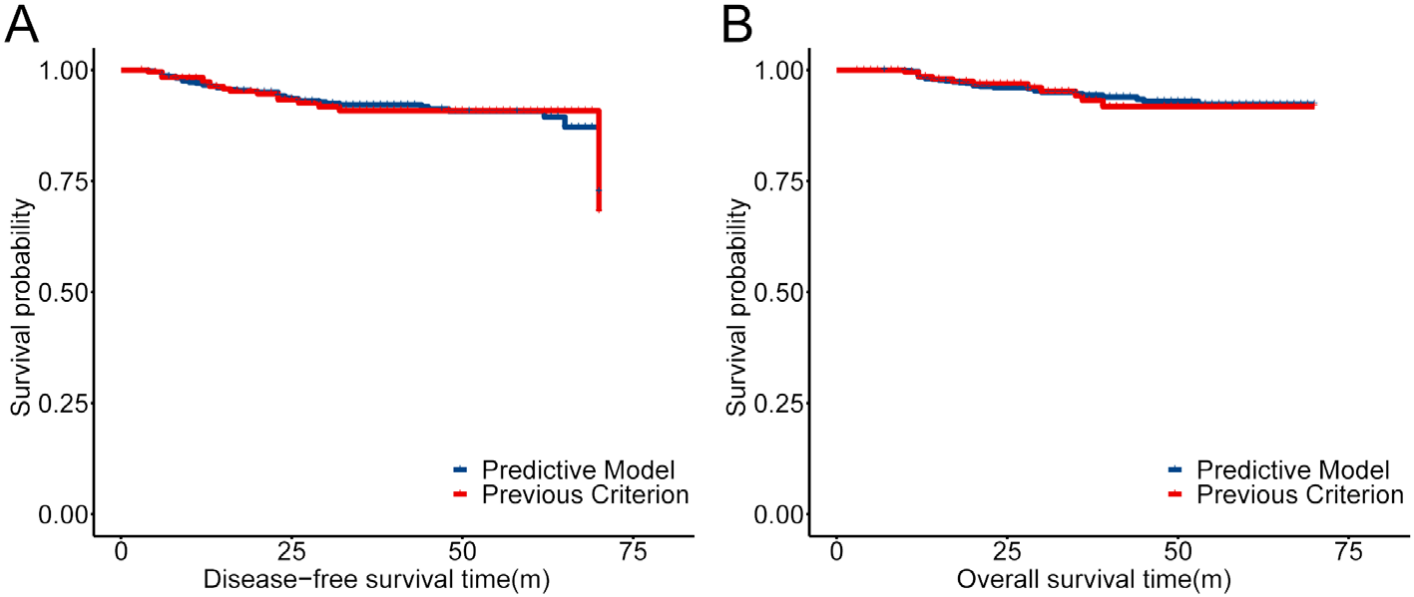
Comparison of the prognosis for the low-risk group defined by our predictive model and previous criteria reported (13). **(A)** KM curves of DFS for the low-risk group defined by our predictive model and previous criteria reported. **(B)** KM curves of OS for the low-risk group defined by our predictive model and previous criteria reported.

Considering the extremely poor prognosis of the occurring ovarian metastases, the low-risk group, predicted by the constructed model, who were recommended to preserve their ovaries, must be guaranteed to be secure. After redefining the whole population into low- and high-risk groups with the predictive model, the low-risk group included 822 patients who were recommended to preserve their ovaries, increasing the OP population screened by the current criteria by 3.21-fold (**Table 6**). The 5-year DFS and OS of the low-risk group had no difference compared with the patients with ovary preservation screened by the current criteria (**Figure 6**), with OM in the low-risk group at only 0.24% and the high-risk group at 16.96% in the total population, which proved the effectiveness and security of this predictive model.

**Table 6:** The evaluation of the total population with the predictive model.

	OM			OP		
	No	Yes	p-Value	No	Yes	p-Value
High risk	93 (10.2)	19 (90.5)	<0.001	102 (15.3)	10 (3.8)	<0.001
Low risk	820 (89.8)	2 (9.5)		566 (84.7)	256 (96.2)	
Total	913	21		668	266	

## Discussion

Patients with early-stage ADC usually have optimistic oncological outcomes, with the incidence of OM at 0.5%–11% (7, 8, 13, 19). In our study, patients with adnexectomy showed the rate of OM at 3.14%, which is consistent with previous reports; however, bias might occur as low-risk younger patients selected OP during surgery. Concurrently, the prognosis of patients with OM seemed quite poor, with a 5-year OS rate at 28%. Our predictive model could precisely predict ovarian metastasis efficiently and potentially increased the young patients who could safely preserve their ovaries by 3.21-fold compared with current selective criteria.

OP could remarkably benefit young patients not only to retain fertility but also improve their quality of life through saving their ovarian endocrine function. Once ovaries are removed, patients sharply seek iatrogenic menopause, which could lead to severe menopausal symptoms, including early hot flashes, vaginal atrophy, an increased risk of cardiovascular disease, osteoporosis, hip fracture, Alzheimer’s disease, and mental health disorders (20).

Most of the parameters in the previous selection criteria mainly depend upon biopsy pathological results and imaging evidence. LNM status mainly relied on the pre-operative imaging test; however, it was reported that the preoperative predictive accuracy of LNM by PET-CT was only approximately 70% (21). Additionally, the status of LVSI reported by biopsy or LEEP pathological examination was as low as 11.6% from our database. Therefore, the efficacy of the selection criteria for OP in ADC patients could be impaired owing to the capabilities of imaging, pathology, and other related departments. With the evidence from this study, we propose a new criterion for OP in ADC or ASC patients as shown in **Table 7**. The critical parameter in this criterion is the cutoff value of OM, which could be adjusted by the clinician for an individual patient with ADC. Here in our population, the OM rate in the low-risk population was 0.24% and 16.96% in the high-risk group, which indicated that patients with OM risk lower than 0.24% were recommended for OP, and those with higher than 16.96% should receive bilateral ovary resection. The decision should be made by the individual patient, with an OM rate between the two values, and her doctor. It is difficult to confirm a reasonable cutoff value between the two. The NCCN Cervical Cancer Guidelines 2023 recommended adjuvant therapy after surgery for patients according to a novel histology-specific nomogram for predicting cervical cancer recurrence risk (22) in which a cutoff recurrence risk of 15% was suggested. However, prognosis with OM is extremely poor based on our analysis (**Figure 2D**), so stricter cutoff for preserving the ovaries should be considered, which should be greatly lower than 15%.

**Table 7:** Updated selection criteria for OP recommended.

Preoperative patients’ characteristics
–Premenopausal women
–Patients who desire to retain ovarian function
–No familial predisposition to ovarian cancer
–OM rate calculated based on preoperative examination ≤ cutoff value*
Intraoperative findings
–No extra-uterine spread except LNM
–Normal appearance of ovaries
–OM rate calculated based on pre- and intra-operative findings ≤ cutoff value
Postoperative pathology
–OM rate ≤ cutoff value, close surveillance according to the routine of cervical cancer follow-up

*The threshold for ovarian metastasis rate to have BSO performed could be individualized and decided with the gynecology oncologist and the patient together.

A multidisciplinary team should be summoned to implement this predictive model; especially, the imaging and pathology departments, which might be limited in resource-poor areas, should be consulted when making decisions. Additionally, it should be emphasized that patients could be assessed as low-risk group pre- and intra-operation, while upgraded as high-risk for OM post-operation, which required another operation to remove the ovaries. These risks should be carefully communicated with patients before OP surgery using this predictive model.

Our study constructed a prediction model for ovarian metastasis in patients with cervical adenocarcinoma/adenosquamous carcinoma for the first time to the best of our knowledge. Current clinical researches are more concerned on how to identify the population with a high risk of OM and if the ovaries preserved have a higher potential of subsequent recurrence than other organs. The OM risk reported previously has been inconsistent. Ritsu Yamamoto et al. found that the metastatic rate of adenocarcinoma was as high as 10.2% (10/98 = 10.2%) (7), after analyzing 631 cases at IB–IIIB treated with radical hysterectomy + pelvic lymph node dissection, for which preservation of the ovaries for patients with ADC should not be recommended. However, nine cases in this study were staged at IIB or IIIB with postoperative pathology, which should be excluded from this study, and surgery should not be recommended. The rate of ovarian metastasis in this study should be amended to 1/52 = 1.92% considering this bias, which is similar to that of other reports.

Cheng et al. reviewed 10 studies on ADC, staged at I–II with surgical treatment, by meta-analysis, with sample sizes of 5,075, and 1,199 who had their ovaries preserved, and 3,867 had their ovaries removed. The overall metastatic rate was 3.61% in patients with ovaries removed, and there was no significant difference in prognosis between patients with preserved ovaries and those with ovaries removed [OS (OR 1.00, 95% CI 0.64–1.56, I2 = 25.7%), PFS (OR 0.98, 95% CI 0.57–1.66, I2 = 0%)] (8). Meanwhile, 19 investigations were reviewed to analyze the potential risk factors for OM to help clinically guide us for ovarian preservation, and the main risk factors included were FIGO stage, tumor size, DSI, parametrial invasion, corpus uteri invasion, LNM, vaginal invasion, and LVSI (8). Similarly, these factors were also identified in our model, and we further quantified their ORs for potential ovarian metastasis (**Figure 3B**) and incorporated them into the prediction model for ovarian metastasis.

For the second concern raised here, Giovanni Scambia et al. concluded that the rate of ovarian recurrence in cervical cancer patients stayed at a relatively low level (0%–1.3%) through a systematic literature review (13). Further subtyping the study cohort, patients with ADC (n = 196) were found with no ovarian metastasis or subsequent secondary ovarian cancer (13). Overall, ovarian preservation was recommended in patients with cervical adenocarcinoma at early stage if with no risk factor. However, this is uneasy to follow in practice as no clear guidance was recommended so far; the only standard we searched was to take all risk factors equally in contribution for OM, and ovarian preservation should not be considered if one risk factor was identified (12). Our study successfully quantified the contribution of each risk factor to OM to avoid unnecessary ovarian removal in young patients to improve their life quality.

Although our predictive model is optimized through machine learning, it still has some limitation. First, it is based on a single-center, retrospective study, and a further multi-center prospective randomized control trial is needed to help validate the model. Second, some parameters in our model require high-quality intraoperative frozen pathological reports in clinical setting. Risk factors, such as fallopian tube metastasis, UCI, and LNM (lymph nodes with suspicious preoperative imaging or intraoperative microscopic metastasis), need intraoperative frozen pathology to detect and help make a clinical decision if OP could safely be performed. Additionally, our model is based on the WHO 2014 pathological classification, while a new classification system (International Endocervical Adenocarcinoma Criteria and Classification, IECC) was established in 2018 (23), which had been adopted in some medical centers. Therefore, they can consider selectively replacing the high-risk pathological types with non-HPV-associated (NHPVA) cervical adenocarcinoma when using our predictive model, as the high-risk pathological types in our study included gastric type, MDA, serous type, and signet ring cell type. Signet ring cell type is very rare, so it can be considered alone regarding OP.

## Conclusion

Ovarian preservation is remarkably important for young patients with ADC to maintain their life quality, and concurrently, patients with OM have extremely poor prognosis, so the individual decision of whether to preserve the ovaries before surgical treatment should be accurately made. Our study constructed a precise predictive model through machine learning with a decent sample size of 934 patients with ADC, which could potentially reduce 60% of young patients with ADC to safely preserve their ovaries compared with the current selective criteria.

## Data Availability

The original contributions presented in the study are included in the article/**Supplementary Material**. Further inquiries can be directed to the corresponding authors.

## Supplementary material

Supplementary Figure 1
**(A)** LASSO coefficient profiles of the 13 key clinicopathological variables for the prediction of ovarian metastasis in the training cohort. **(B)** Tuning parameter selection by 10-fold cross-validation in the LASSO model of training cohort. The partial likelihood deviance was plotted against log (lambda/λ), and λ was the tuning parameter. The partial likelihood deviance values are shown, and error bars represented s.e. The dotted vertical lines showed the optimal values through minimum criteria and 1−s.e. criteria. **(C)** Tenfold cross-validation showed that three variables could minimize the error of optimum subset regression analysis in the training cohort. The “bestglm” R package was used.
