# Supplementary figures and images for: How do we safely preserve ovaries in patients with cervical adenocarcinoma: risk factors and predictive models
Source: Front Oncol. 2024 Oct 30;14:1464565. doi: 10.3389/fonc.2024.1464565 (PMC11557467; doi:10.3389/fonc.2024.1464565)

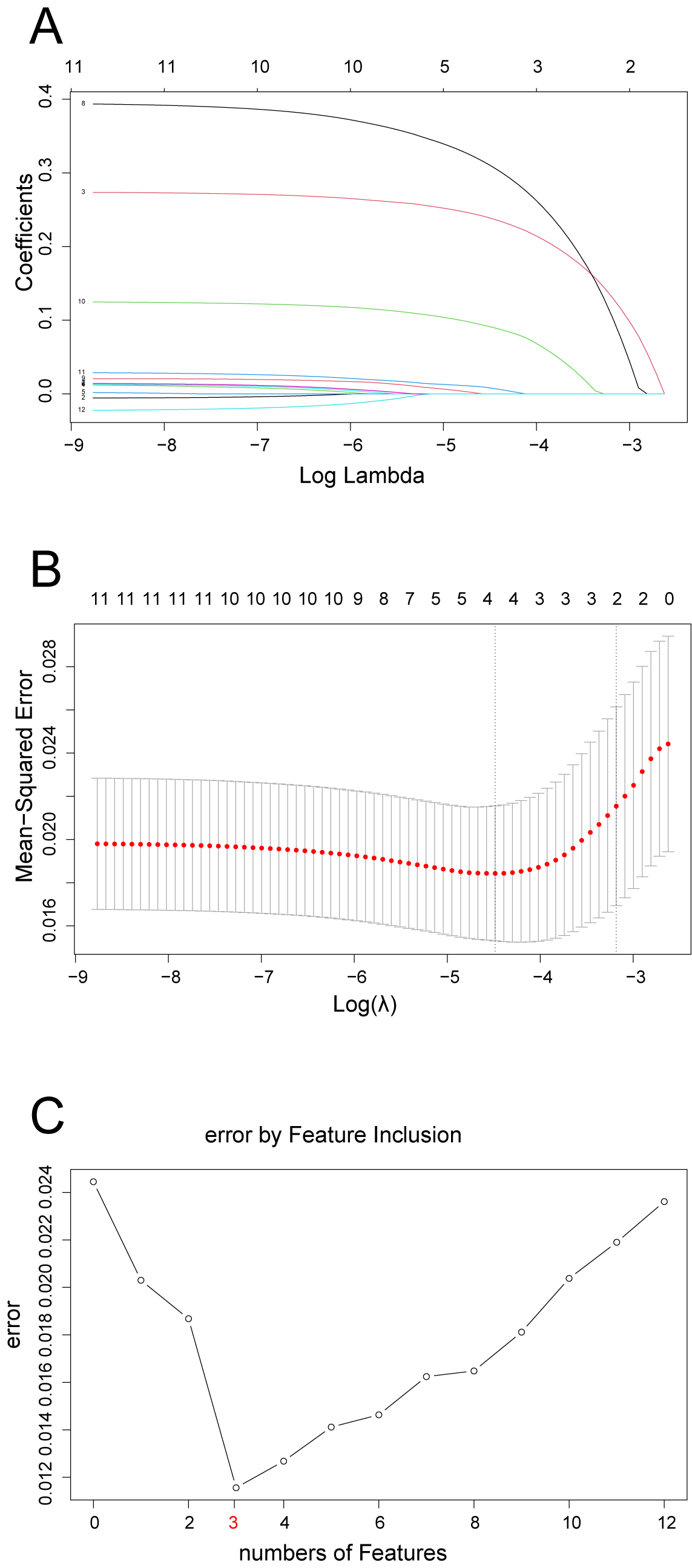

Supplement: Supplementary Figure 1 — (A) LASSO coefficient profiles of the 13 key clinicopathological variables for the prediction of ovarian metastasis in the training cohort. (B) Tuning parameter selection by 10-fold cross-validation in the LASSO model of training cohort. The partial likelihood deviance was plotted against log (lambda/λ), and λ was the tuning parameter. The partial likelihood deviance values are shown, and error bars represented s.e. The dotted vertical lines showed the optimal values through minimum criteria and 1−s.e. criteria. (C) Tenfold cross-validation showed that three variables could minimize the error of optimum subset regression analysis in the training cohort. The “bestglm” R package was used. [file Image1.tif]
